# Miss Ethel M. Warner and Mr. Lawson Tait

**Published:** 1892-12-17

**Authors:** Lawson Tait


### Miss Ethel M. Warner and Mr. Lawson Tait.

By Lawson Tait, F.R.C.S.

My attention has been drawn to a letter in your columns from Miss Ethel M. Warner, who doubtless was the contributor of a letter on the same subject to the *Times* some weeks ago, to which the editor of that paper did not permit me a reply.

Miss Warner ought not to adopt as a privilege of her sex the use of such language as appears in her letter, for the accusation of "making false statements" ought to be left to that sex to whom I could fittingly reply. Permit me, however, to state that her knowledge of the subject is extremely rudimentary in every aspect, that the proper position of Dr. Macewen was given in the columns of the *Times* of December 26th, 1884, and January 3rd and 13th, 1885, at the time the discussion took place, with Dr. Macewen's full consent and approbation. As to Mr. Rickman Godlee's present opinion I can only refer to the one which he gave also at the time of that discussion at a meeting of the Royal Medical and Chirurgical Society on May 12th, 1885, and contrast it with that attributed to him now by Miss Warner. It is quoted from the pages of the *British Medical Journal* of May 16th, of the same year, and it expresses the opinion which I myself had at the time, and which I have seen no occasion to alter. Mr. Godlee's words are as follows :—" Professor Ferrier's and Mr. Horsley's experience furnished an *argumentum ad Simiam*, but he could not feel justified in admitting that it applied exactly to man."

It is most strange to see how those on the side of this question who share the views of Miss Warner can be found, like Sir Andrew Clark, to express one opinion on the subject in 1883, and, without any reason given for the change, to enunciate something absolutely the reverse in 1889. These extraordinary alterations require very complete justification.

